# Strain specific protective immunity induced by pre-erythrocytic infection of toque monkeys with *Plasmodium cynomolg*

**DOI:** 10.1186/1475-2875-9-S2-P58

**Published:** 2010-10-20

**Authors:** Wathsala Wijayalath, Sandie Cheesman, Jagath Rajakaruna, Sudath Weerasinghe, Gamini Kapilananda, Kamal Gamage, Kamal Perera, Shiroma Handunnetti, Richard Carter, Sisira Pathirana

**Affiliations:** 1 Malaria Research Unit, Dept of Parasitology, Faculty of Medicine, University of Colombo, Colombo, Sri Lanka; 2 Animal House, Faculty of Medicine, University of Colombo, Colombo, Sri Lanka; 3 School of Biological Sciences, Institute of Immunology & Infection Research, University of Edinburgh, UK; 4 Institute of Biochemistry, Molecular Biology and Biotechnology, University of Colombo, Colombo, Sri Lanka

## 

Strain specific protective immunity (SSPI) was investigated using two strains of *Plasmodium cynomolgi,* Pc746 and PcCeylon, in toque monkey. Two groups of monkeys were immunized against either Pc746 (n=5) or PcCeylon (n=4), by giving bites with 2-4 sporozoite-infected *Anopheles tessellates* mosquitoes per monkey. Primary blood infection was prevented by simultaneous chloroquine cover and secondary, hypnozoite-induced, blood infection was prevented by treating the monkeys one month later with primaquine. The two immunized groups and a group of unimmunized monkeys (n=4) were given a mixed-strain sporozoite challenge infection, 140 and 100 days respectively after immunization. Parasite DNA was collected for 5-8 consecutive days after parasitaemia reached 0.05% or above. The proportions of the two parasite strains in these samples were quantified using a Pyrosequencing^TM^ assay based on SNPs in MSP1 and CSP genes. In subsequent blood infections in immunized monkeys the earliest recorded proportion of parasites of an immunizing strain was significantly lower than its proportion in monkeys immunized against a heterologous strain (P=0.014 and 0.027 for the two SNPs) and the proportions of an immunizing strain tended to decline during the period of sample collection. These results show that a parasite strain specific protective immunity to *P. cynomolgi* was indcued following a sporozoite induced pre-erythrocytic infection. This immunity may have been directed against the liver stages or against the blood stage parasites, or against both

